# PROTAC-Surufatinib Suppresses Pancreatic Neuroendocrine Neoplasms Progression by Inducing Ferroptosis through Inhibiting WNT/β-catenin Pathway Mediated by HMOX1

**DOI:** 10.7150/ijbs.106357

**Published:** 2025-03-19

**Authors:** Bingyan Xue, Lijun Yan, Mujie Ye, Danyang Gu, Jianqiang Qian, Na He, Ping Hu, Feiyu Lu, Xintong Lu, Min Liu, Lin Xu, Jianan Bai, Yan Wang, Guoqin Zhu, Qiyun Tang

**Affiliations:** 1 Department of Geriatric Gastroenterology, Neuroendocrine Tumor Center, Jiangsu Province Hospital, The First Affiliated Hospital of Nanjing Medical University, Institute of Neuroendocrine Tumor, Nanjing Medical University, Nanjing, China.; 2 China pharmaceutical university school of traditional Chinese pharmacy, Nanjing, China.; 3 Digestive Endoscopy, Jiangsu Province Hospital, The First Affiliated Hospital of Nanjing Medical University, China.; 4 Department of Gastroenterology, The Friendship Hospital of Ili Kazakh Autonomous Prefecture, Ili State 835000, China.

**Keywords:** PROTAC, Surufatinib, Pancreatic neuroendocrine neoplasms, Ferroptosis, HMOX1, WNT/β-catenin signaling pathway

## Abstract

The small-molecule targeting drug Surufatinib is a new strategy for pancreatic neuroendocrine neoplasms (pNENs). However, the adverse reactions of Surufatinib should not be ignored in clinical practice. Based on PROTAC technology, we developed a novel tyrosine kinase (TK) degrader PROTAC-Surufatinib (hereinafter referred to as P-Surufatinib). This study was designed to investigate the effects and underlying mechanism of P-Surufatinib on pNENs. *In vitro*, we revealed that P-Surufatinib could more effectively inhibit proliferation and angiogenesis, and degrade target proteins in pNENs cells than Surufatinib. The transcriptome sequencing revealed that HMOX1 was the key molecule of P-Surufatinib to inhibit proliferation in pNENs. It was demonstrated that HMOX1 was lowly expressed in pNENs, and P-Surufatinib could up-regulate the expression of HMOX1 in pNENs. Mechanistically, P-Surufatinib inhibited pNENs progression by inducing ferroptosis through the suppression of HMOX1 mediated WNT/β-catenin signaling pathway. *In vivo*, P-Surufatinib could obviously suppress the growth of subcutaneous tumors in nude mice and enhance the expressional level of HMOX1 in tumorous tissue. In summary, our findings reveal that P-Surufatinib can suppress pNENs progression via inducing ferroptosis through up-regulating the expressional level of HMOX1 by inhibiting WNT/β-catenin signaling pathway, which provides a novel treatment method for pNENs.

## Introduction

Neuroendocrine neoplasms (NENs) are a general term for highly heterogeneous tumors originating from neuroendocrine cells distributed throughout the body, which mainly occur in the digestive tract and pancreas^1^. Over the past few years, the overall incidence of pNENs has been increasing worldwide, accounting for over 3% of pancreatic tumors^2^. The most effective treatment method for pNENs is radical removal. Nevertheless, the pNENs can often be diagnosed in the late stage due to the absence of significant clinical symptoms in the early stages. Alarmingly, more than 60% of patients with pNENs present with distant metastases at their initial diagnosis^3^, ^4^. Molecularly targeted treatment, biologic treatment and chemotherapy are the main treatment options for patients with pNENs that cannot be surgically removed or are accompanied by distant metastases^5^, ^6^.

Surufatinib is a newly developed oral tyrosine kinase inhibitor (TKI) by Chinese experts, which can specifically target to VEGFR, FGFR1 and CSF1R kinases, and has dual effects of anti-angiogenesis and immunomodulation, thereby exerting synergistic anti-tumor effect. In November 2019, Surufatinib was granted FDA orphan drug designation for pNENs. In December 2020 and June 2021, Surufatinib was approved as a monotherapy for unresectable locally advanced or metastatic, well-differentiated extrapancreatic NENs and pNENs in China, respectively. Several clinical trials have shown that Surufatinib has a good anti-tumor effect in the treatment of patients with extra-pancreatic and pNENs^7^-^10^. However, the clinical application of Surufatinib indicated that the adverse reactions such as hypertension, albuminuria and kidney injury should not be ignored^7^, ^11^-^14^. Consequently, it is urgently required to look for a novel method to enhance the targeting efficiency of Surufatinib and thus reduce its usage dose and frequency, so as to improve the efficacy of TKI and reduce the incidence of adverse reactions.

Proteolysis-targeting chimera (PROTAC) has emerged as a highly effective approach for degrading proteins, which is widely employed as a biological means in developing a molecular therapeutic drug with obvious clinical implication^15^-^18^. A typical PROTAC is a bifunctional molecule consisting of a target-specific ligand and an E3 ubiquitin ligase ligand that are connected by a cross-linker. Therefore, this bifunctional design enables PROTAC to recruit E3 ligase onto target proteins to induce their ubiquitination and degradation through the proteasome pathway^15^. This technique may provide a novel approach for degrading TK and improving the targeting efficiency of Surufatinib. In this study, Surufatinib was selectively used as a ligand because of its excellent selectivity and comparatively low molecule weight. The other ligand pomalidomide was selectively used due to its high binding affinity to E3 ligase thereby a corresponding series of PROTACs were developed, of which, P-Surufatinib exhibited exceptional pharmacodynamic characteristics, such as facilitating the degradation of TK and exerting a better anti-tumor effect on pNENs cells compared with Surufatinib, which primarily attributed to ferroptosis-mediated cellular death.

Ferroptosis is a novel pattern of programmed cellular death, which is obviously different from apoptosis and pyroptosis, and has characteristics such as increased levels of reactive oxygen species (ROS), lipid peroxidation (LPO) and iron. Ferroptosis exerts an essential effect in the development of tumors^19^-^21^. However, the specific mechanism of ferroptosis in pNENs remains to be further elucidated. Hemoxygenase 1 (HMOX1) is an essential enzyme in the catabolism of heme, it can decompose heme and release Fe^2+^, which can produce ROS via the Fenton reaction and thus promote the generation of LPO, further disrupt iron homeostasis and induce ferroptosis^19^, ^22^. However, there have been few reports on the effect of HMOX1 on ferroptosis of pNENs cells.

This study was designed to investigate a novel method to promote the targeting efficiency of Surufatinib through the use of PROTAC, which can exert effects in TK degradation and inducing ferroptosis in pNENs cells. This study will offer a new treatment option for patients with pNENs.

## Methods

**Cellular cultivation:** The human pancreatic nestin-expressing ductal cell line (HPNE) was provided by American Type Culture Collection (CBP60857). The human pNENs cell line QGP-1 was provided by Japanese Collection of Research Bioresources cell bank (JCRB0183). The BON-1 cell line was donated by Prof. Xianjun Yu from Affiliated Cancer Hospital of Fudan University. The human umbilical vein endothelial cell line (HUVEC) was provided by the ATCC (Cat. No. CRL-1730). HPNE, QGP-1 and HUVEC were cultivated in RPMI-1640 (Gibco, America) while BON-1 were cultivated in DMEM/F-12 (1:1) (Gibco, America), which was added with 10% FBS [Yeasen Biotechnology (Shanghai) Co., Ltd., China] and 1% penicillin/streptomycin solution (P/S, Thermo scientific HyClone, America). All these cell lines were cultivated in humidified CO_2_ incubators at 37 °C.

**Constructing stable transfected cell lines:** We constructed HMOX1 overexpressing plasmids in the PLVX vector [Genomeditech (Shanghai) Co., Ltd., China], and HMOX1-knockdowning plasmids in the PLKO1 vector [Genomeditech (Shanghai) Co., Ltd., China]. The 293 T cells were transfected with lentiviruses using PEI MAX (Polysciences, America). Stable transfected cells were obtained following virus infection and filtration through the addition of puromycin to the media. See specific shRNA targets in Table S1.

**Quantitative real-time PCR (qRT-PCR):** Total RNAs were extracted from pNENs cells by using the total RNA rapid extraction kit (TRIzol method). The genomic DNA was eliminated using 5× gDNA digester [Yeasen Biotechnology (Shanghai) Co., Ltd., China], and the reaction was maintained for 2 min at 42°C, and then cDNAs were synthesized after the 4xHifair^®^ III SuperMix plus [Yeasen Biotechnology (Shanghai) Co., Ltd., China] was added for reverse transcription at 37°C for 15 min and 85°C for 5 s. Roche instrument and SYBR Green PCR Mastermix [Yeasen Biotechnology (Shanghai) Co., Ltd., China] were applied to perform qRT-PCR; the procedure was as follows: 95°C for 5 min, 35 cycles at 95°C for 30 s, 58°C for 30 s, 72°C for 30 s. β-actin serves as an internal reference. All primers used in this work were displayed in Table S2. 2^-△△ct^ method was used for data analysis, and GraphPad Prism 9.0 was used for data visualization.

**Western blotting (WB) experiment:** All proteins were extracted using NP-40 Lysis Buffer comprising 1% 100 mM Phenylmethanesulfonyl fluoride(PMSF, Beyotime Biotechnology) for 30 min, and soaked in 1×loading buffer at 100 °C for 10 min. Equal quantities of proteins were electrically separated using 10% SDS-polyacrylamide gel (Suzhou NCM Biotechnology Co., Ltd, China) and then transferred onto nitrocellulose membranes (Millipore, America). The membranes underwent blocking using 8% milk TBST (4 g skim milk powder and 50 ml TBST) for 2 h and overnight incubation at 4°C using primary antibodies after being washed three times using TBST solution (Wuhan Sevicebio Biotechnology Co., Ltd, China), followed by three washings again using TBST solution, 10 min per time. Afterwards, the band was treated using anti-rabbit or anti-mouse IgG at normal temperature for 1 h. Details regarding the antibodies can be found in Table S3. The enhanced chemiluminescence (NCM Biotech) was added to develop images using Image-Lab 6.1(Bio-Rad Laboratories, America).

**Immunofluorescent experiment:** The 24-well plates were seeded with 2×10^4^ cells, which underwent overnight incubation using 200 μL of hybridization buffer and washing thrice using PBST and fixation in 4% paraformaldehyde solution. Thereafter, the cells underwent 15 min of incubation using 0.5% Triton X-100, 30 min of blocking using goat serum and 60 min of incubation using primary HMOX1 antibodies, respectively. After that the cells underwent washing thrice using PBST and then1 h incubation at normal temperature using Coralite488-conjugated goat anti-rabbit IgG (H+L) (Proteintech Group, America). After 30 min of incubation using Hoechst 33342 staining, a random image selection was carried out under fluorescent microscopy (Olympus Optical Co., Ltd., Japan).

**Cell Counting Kit-8 (CCK-8) experiment:** The growth of QGP-1 and BON-1 cells was assessed with CCK-8 experiment. 5×10^3^ QGP-1 cells or 5×10^3^ BON-1 cells were seeded in each well of a 96-well plate containing 100 μL of medium. Each well was added with 10 μL of CCK-8 solution (Vazyme Biotech Co., Ltd., China) and cultivated in an incubator for 2 h at 37 °C respectively after 0, 24, 48, and 72 hours of cell cultivation, respectively, and the optical densities were measured at a wavelength of 450 nm with microplate readers.

**Colony-forming experiment:** In the colony-forming experiment, a total of 1×10^3^ QGP-1 or 1×10^3^ BON-1 cells were seeded into 6-well plates for 14 days of cultivation in complete media, followed by 30 min of cellular fixation using 4% paraformaldehyde and 30 min of staining using 0.25% crystal violet.

**EdU incorporation experiment:** The cells seeded in a 96-well plate were processed at 37°C for 2 h using 50 µM 5-ethynyl-2'-deoxyuridine (EdU) (Guangzhou RiboBio Biotechnology Co., Ltd, China), followed by 30 min of fixation using 4% paraformaldehyde. After being permeabilized using 0.5% Triton X-100, the cells were treated with 1× Apollo reaction cocktail for half an hour. Finally, the cell nuclei were stained using Hoechst 33342 for half an hour., and the fluorescent microscopy was used to visualize and save images.

**Tube formation experiment:** The experimental equipment such as 96-well culture plates and sterile pipette head were pre-cooled. 50uL of Matrigel matrix (Corning, America) was placed into the 96-well plate for 30 min of incubation at 37°C to turn into a gel. HUVEC cells at logarithmic phase of growth were inoculated on the gel surface with a cell density of 3×10^4^/ well for 4-6 hours of incubation. An Olympus inverted microscope was used to take images, which were further quantified by ImageJ software program.

**Lipid peroxidation experiment:** A total of 5×10^5^ QGP-1 or BON-1 cells were seeded into each well of a 48-well plate for overnight culture. Following this, the plate was added with either DMSO or P-Surufatinib for additional 48 h of culture. Subsequently, each well was supplemented with 50 μmol/L C11-BODIPY581/589 (Thermo Fisher, America), and further underwent 20 min of culture at 37 °C, followed three times of washing using PBS. Finally, the microscopy was performed to develop fluorescent images.

**Detection of ROS levels:** DCFH-DA probe was used to measure ROS level. QGP-1 or BON-1 cells were placed in 6-well plates and further assigned into two groups such as control and P-Surufatinib groups, respectively, with an equal quantity of cells in each group. Thereafter, the medium was removed, the cellular washing was repeated two times using PBS, and each plate was supplemented with 10μmol/L DCFH-DA (2mL). Subsequently, 20 min of cellular incubation was performed in a dark room at 37°C, and the DCFH-DA probe was decanted, followed by a thorough washing. Finally, the cells were harvested, and the ROS level within cells was measured using a flow cytometer.

**Iron level measurement:** Cellular iron level was detected by Ferrous Ion Content Assay Kit (BC5415, Solarbio Science & Technology, Beijing, China), according to the manufacture's protocol. Cell samples (1 × 10^7^ cells) were lysed by 1 mL lysate and broken by ultrasound (200 W, last 5 s and interval 5 s, repeat 30 times) and then centrifuged at 10000×g for 10 min. Finally, a microplate reader was used to measure the absorbance at 593 nm.

**Establishment of xenograft mouse models:** A total of 36 four-to-five week-old male BALB/c nude mice were provided by the Animal Center of Nanjing Medical University (Nanjing, China) and raised in a sterile environment. Each mouse was subcutaneously injected with 2×10^6^ QGP-1 or BON-1 cells. Each mouse in the Surufatinib group was administrated by gavage with Surufatinib at a dose of 60 mg/kg/day; each mouse in the P- Surufatinib group was administrated by gavage with P- Surufatinib at a dose of 20 mg/kg/day; each mouse in the P- Surufatinib+Fer1 group was administrated by feeding P- Surufatinib of 20 mg/kg/day and intraperitoneal injection Fer1 of 5 mg/kg/d. Four weeks later, all of the mice were put to death, and the tumors were excised. After treatment, the weight, width and length of the tumors were detected, their images were taken, and then all tumors underwent fixation using 4% paraformaldehyde for subsequent analyses. The formula for calculating the tumor volume (V) was as follow: V = (width^2^ × length)/2. The tumor weigh was precisely detected the tumor-inhibiting rate (TIR) using the formula as follows: TIR (%) = (Wcontrol - Wsample) / Wcontrol × 100. All protocols involving animal experimentation were approved by the Animal Care and Use Committee of Nanjing Medical University.

**Immunohistochemistrical staining:** Tumor tissues were preserved in 4% formaldehyde and then paraffin-embedded. The paraffin-embedded tissues were sectioned into 5μm slices, which were co-cultivated with related antibodies overnight in the humidified room at 4°C, followed by three times of washing and 1 h of incubation with secondary antibodies at normal temperature. Thereafter, DAB solution was used to perform hematoxylin staining. After dehydration, the slices were photographed under optical microscopy (XSP-C204).

**Statistical analysis:** GraphPad Prism 9.0 was used for data analysis, and the unpaired or paired student's *t* test was performed for pairwise comparisons. All data were described as mean ± standard deviation (M±SD). *P* < 0.05 indicated that the difference was statistically significant. Each experiment was repeated at least thrice.

## Results

### Novel TK degrader with Surufatinib as a ligand is synthesized and screened based on PROTAC technique

**Figure 1:**
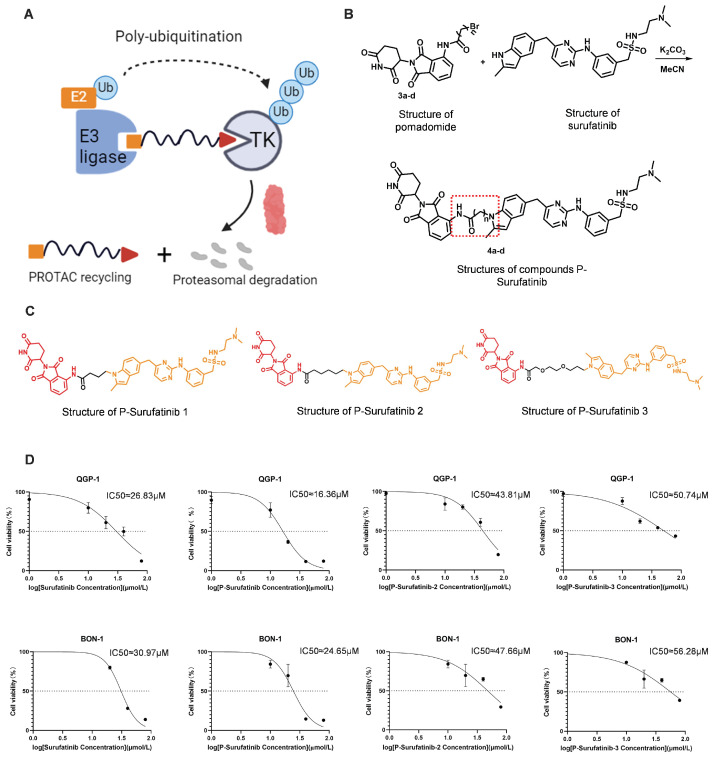
Design strategy for the potent P-Surufatinib. (A) The schematic diagram was drawn for TK protein degradation mediated by PROTAC. (B, C) Pomadomide and surufatinib were used for chemical synthesis of a series of compounds P-Surufatinib 1-3. (D) The half-maximal inhibitory concentration values of Surufatinib and compounds P-Surufatinib 1-3 were assessed in QGP-1 and BON-1 cells.

P-Surufatinib was a compound capable of forming a ternary complex with TK and E3 ligase, which could facilitate the transfer of ubiquitin to TK, leading to its degradation of TK by endogenous proteasomes (Fig. 1A). For designing PROTAC that could target TK, the pomadomide was selectively used as a ligand to bind to E3 ubiquitin ligase. Surufatinib and pomadomide were linked by alkyl chains, thus resulting in multiple compounds such as P-Surufatinib 1-3. The structures of Surufatinib, pomadomide and P-Surufatinib 1-3 are clearly shown (Fig. 1B, C). The more detailed synthesis route is shown in Supplementary file 1. Half-maximal inhibitory concentration (IC_50_) values for Surufatinib, P-Surufatinib 1-3 against pNENs cells were determined using CCK-8 experiment after 48 h of treatment. Then the P-Surufatinib with the best activity and the smallest IC_50_ was selected (Table 1 and Fig. 1D).

### P-Surufatinib can decrease pNENs cell proliferation and angiogenesis via degrading target proteins more significantly than Surufatinib

**Figure 2:**
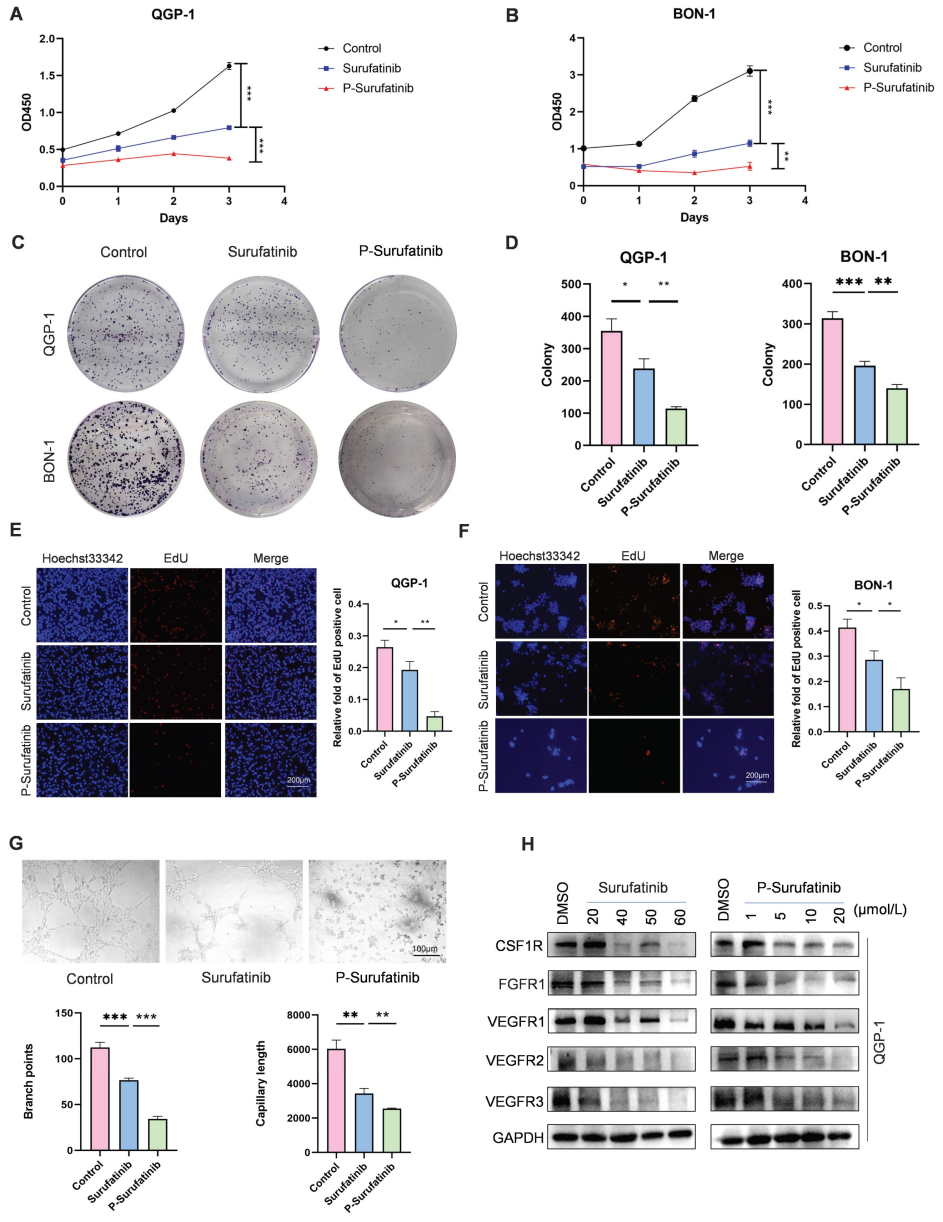
Surufatinib and P-Surufatinib decrease pNENs cell proliferation, inhibit angiogenesis and degrade target proteins. (A, B) CCK-8 assay showed that Surufatinib significantly inhibited the proliferation of pNEN cells, P-Surufatinib was more effective than Surufatinib in inhibiting the proliferation of pNENs. (C, D) The colony formation indicated that P-Surufatinib was more effective than Surufatinib in inhibiting the proliferation of pNENs. (E, F) An EdU assay was performed to explore the effects of Surufatinib and P-Surufatinib on cell proliferation and also displayed P-Surufatinib was more effective than Surufatinib in inhibiting DNA synthesis. Magnification: 200×. (G) Angiogenesis experiments showed that Surufatinib reduced angiogenesis, while P-sufatinib more strongly inhibited angiogenesis. Magnification: 100×. (H) The expression of target protein in pNENs cells treated with different concentrations of Surufatinib and P-Surufatinib for 48h was detected by WB. *P< 0.01, **P < 0.01, ***P < 0.001.

In this study, before the experiments, the QGP-1 and BON-1 cells in Surufatinib group underwent 48 h of treatment with 25 μM and 30 μM doses of Surufatinib, respectively. Meanwhile, the QGP-1 and BON-1 cells in P-Surufatinib group underwent 48 h of treatment with 15 μM and 25 μM doses of P-Surufatinib, respectively. CCK-8 and colony-forming experiments revealed that Surufatinib could obviously inhibit the pNENs cell proliferation. The EdU experiment also indicated that Surufatinib could reduce pNENs cell proliferation. However, P-Surufatinib was more effective in inhibiting the pNENs cell proliferation compared with Surufatinib (Fig. 2A-F). A tube formation experiment was carried out to investigate and compare the effects of Surufatinib and P-Surufatinib on angiogenesis of HUVEC, and the results showed that Surufatinib could suppress angiogenesis, while P-Surufatinib could inhibit angiogenesis more effectively compared with Surufatinib (Fig. 2G). For determining the effect of P-Surufatinib in TK degradation, the expressional levels of VEGFR1/2/3, CSF-1R and FGFR1 proteins were detected and compared between P-Surufatinib group and Surufatinib group. The expressional levels of VEGFR1/2/3, CSF-1R and FGFR1 were decreased more significantly in a dose-dependent pattern in P-Surufatinib group and over 90% target proteins underwent degradation after QGP-1 cells were treated using 20 μM dose of P-Surufatinib (Fig. 2H). Thereafter half-maximal degradation concentration (Dc_50_) values of Surufatinib and P-Surufatinib for different target proteins were detected respectively using Image-J, and it was found that P-Surufatinib was superior to Surufatinib in degrading target proteins (Table 2). Taken together, the above results indicate that P-Surufatinib has stronger anti-proliferative, anti-target proteins and anti-angiogenesis effects on pNENs cells compared with Surufatinib.

### P-Surufatinib can inhibit the progression of pNENs via up-regulating HMOX1

**Figure 3:**
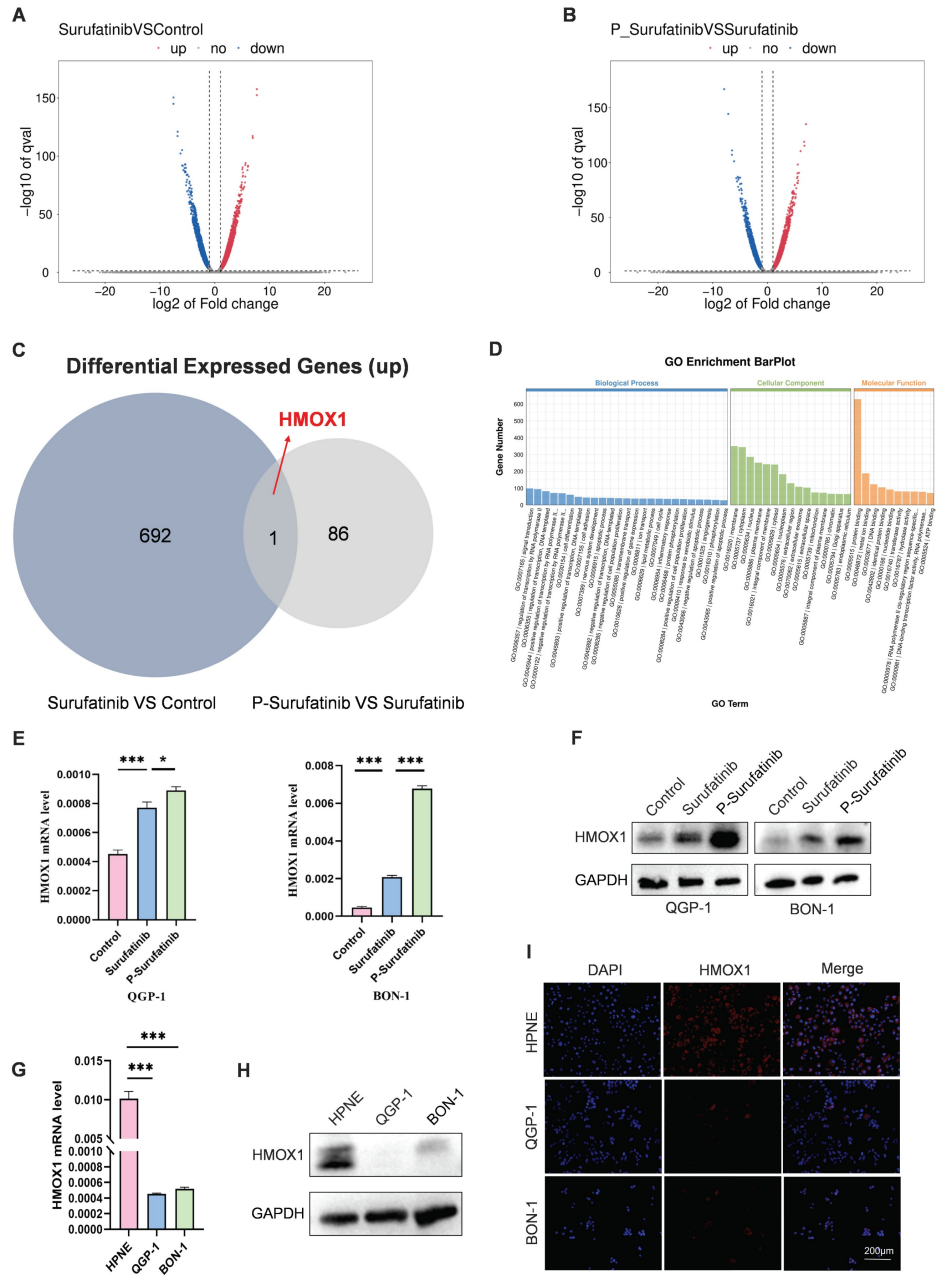
P-Surufatinib affects multiple biological processes. (A-C) 693 genes were upregulated after 48h treatment with Surufatinib and DMSO in QGP-1 cells, while 87 genes were upregulated after 48h treatment with P-Surufatinib and Surufatinib in QGP-1 cells, HMOX1 was the only gene that was found to be up-regulated in the two comparisons mentioned above. (D) GO analysis for RNA-seq results showed that P-Surufatinib affects multiple biological processes. (E, F) qRT-PCR and WB showed that P-Surufatinib upregulated HMOX1 expression in pNENs cells. (G-I) qRT-PCR, WB and immunofluorescence indicated that HMOX1 expression was lower in pNENs cells than in HPNE. Magnification: 200×. *P< 0.01, **P < 0.01, ***P < 0.001.

RNA-seq assays were carried out to determine the mechanism for the effects of Surufatinib and P-Surufatinib on pNENs progression. A lot of differentially expressed genes (DEGs) were measured in QGP-1 cells processed using Surufatinib, P-Surufatinib and DMSO, respectively (Fig. 3A, B). A total of 693 genes were up-regulated in QGP-1 cells after 48 h of treatment with Surufatinib compared with DMSO, while 87 genes were up-regulated in QGP-1 cells after 48 h of treatment with P-Surufatinib compared with Surufatinib. HMOX1 was the only gene that was found to be up-regulated in the two comparisons mentioned above (Fig. 3C). Gene ontology (GO) enrichment analyses showed that P-Surufatinib might play an important role in many biological processes (Fig. 3D).

To determine the variation in the gene expression, qRT-PCR and WB were used to evaluate mRNA and protein expression levels, respectively. The results indicated that the mRNA and protein expression levels of HMOX1 were up-regulated in Surufatinib group and much more up-regulated in P-Surufatinib group (Fig. 3E, F).

The effect of HMOX1 on pNENs was assessed by qRT-PCR and WB, it was found that the expression level of HMOX1 was lower in pNENs cells than in normal human pancreatic cells (Fig. 3G, H), and the immunofluorescent experiment also revealed the distribution of HMOX1 in different cells (Fig. 3I). The above findings demonstrated that the expressional level of HMOX1 was low in pNENs, while P-Surufatinib could up-regulate the expressional level of HMOX1 in pNENs.

### Overexpressed HMOX1 can suppress the proliferation of pNENs cells

**Figure 4:**
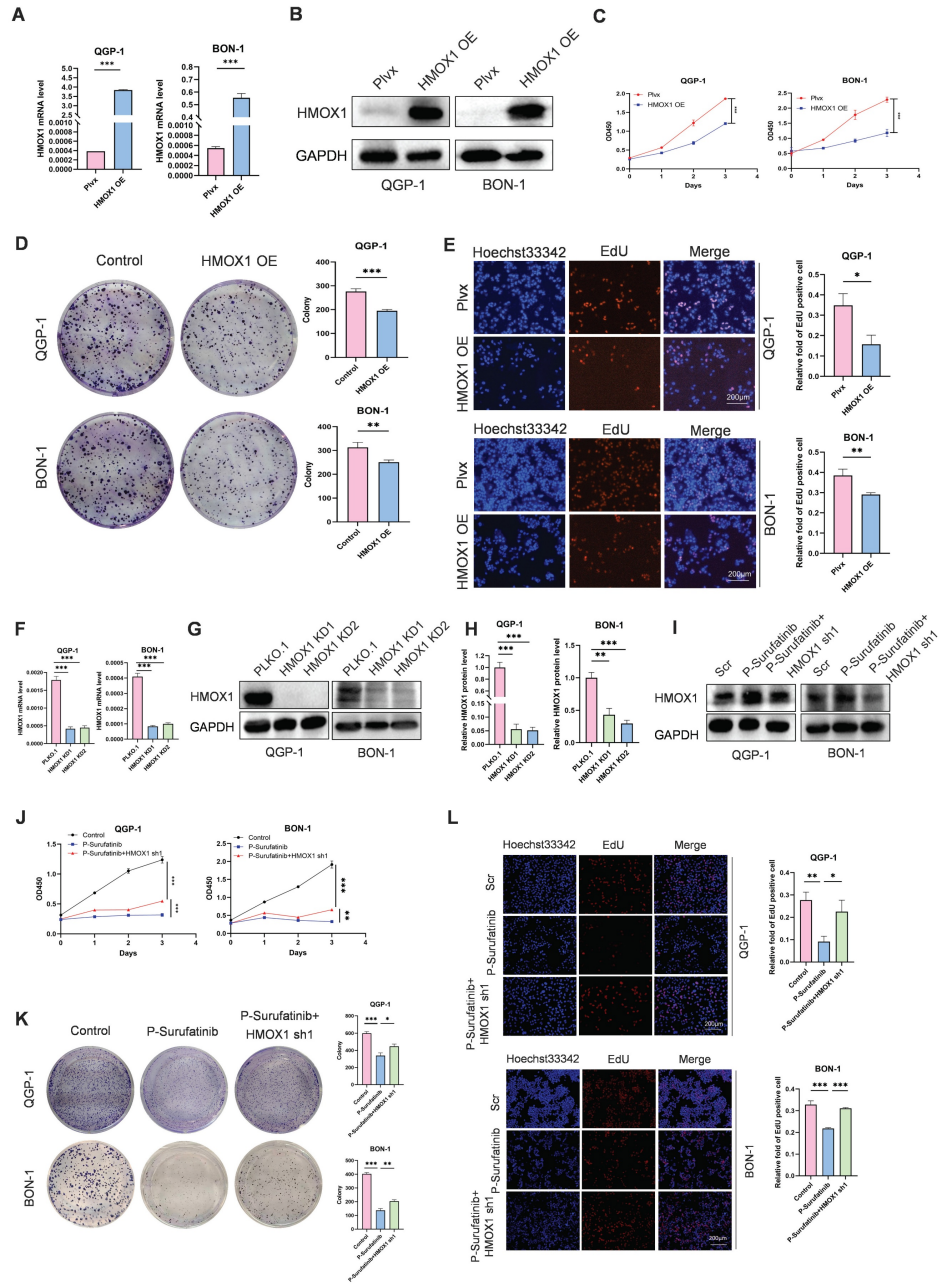
P-Surufatinib suppresses pNENs cell viability by upregulating HMOX1. (A, B) pNENs cell lines stably overexpression of HMOX1 was constructed and assayed by qRT-PCR and WB. (C) CCK-8 assay showed that overexpression of HMOX1 inhibited pNENs cell proliferation rate. (D) The colony formation indicated that overexpression of HMOX1 inhibited pNENs cells proliferation. (E) Overexpression of HMOX1 significantly inhibited DNA synthesis. Magnification: 200×. (F-H) pNENs cells stably knockdown of HMOX1 were constructed and assayed by qRT-PCR and WB. (I) Immunoblots of pNENs cells treated with P-Surufatinib for 48h and then HMOX1 knockdown. (J-L) CCK-8, colony formation, and EdU incorporation assays showed that knockdown of HMOX1 reversed the tumor-suppressive effects of P-Surufatinib* in vitro*. Magnification: 200×. *P< 0.01, **P < 0.01, ***P < 0.001.

To explore the effect of HMOX1 on pNENs, the QGP-1 and BON-1 cell lines with stably overexpressed HMOX1 were established, and their transfection efficiencies were detected by qRT-PCR and WB (Fig. 4A, B). CCK-8 and colony-forming experiments indicated that the proliferation of cells with stably overexpressed HMOX1 was significantly suppressed (Fig. 4C, D). An EdU experiment was conducted for determining the role of HMOX1 in affecting the proliferation of cells (Fig. 4E). The findings confirmed that overexpressed HMOX1 could inhibit the proliferation of pNENs cells.

### Inhibition of HMOX1 can block the effects of P-Surufatinib on proliferation in pNENs

To investigate whether P-Surufatinib could hinder the progression of pNENs through the regulation of HMOX1 expression, and whether knockdowning the expression of HMOX1 could block the tumor-suppressing effect of P-Surufatinib, the genetic rescue experiment was conducted in pNENs cells. The short hairpin RNAs (shRNAs) were applied for knockdowning the expression of HMOX1 in P-Surufatinib-treated pNENs cells, and their efficiencies were detected using qRT-PCR and WB (Fig. 4F-I). All CCK-8, colony-forming and EdU experiments confirmed that knockdowning the expression of HMOX1 would obviously reverse the suppressive effect of P-Surufatinib on pNENs cells proliferation (Fig. 4J-L).

### P-Surufatinib can up-regulate the expression of HMOX1 to induce ferroptosis for suppressing pNENs progression

**Figure 5:**
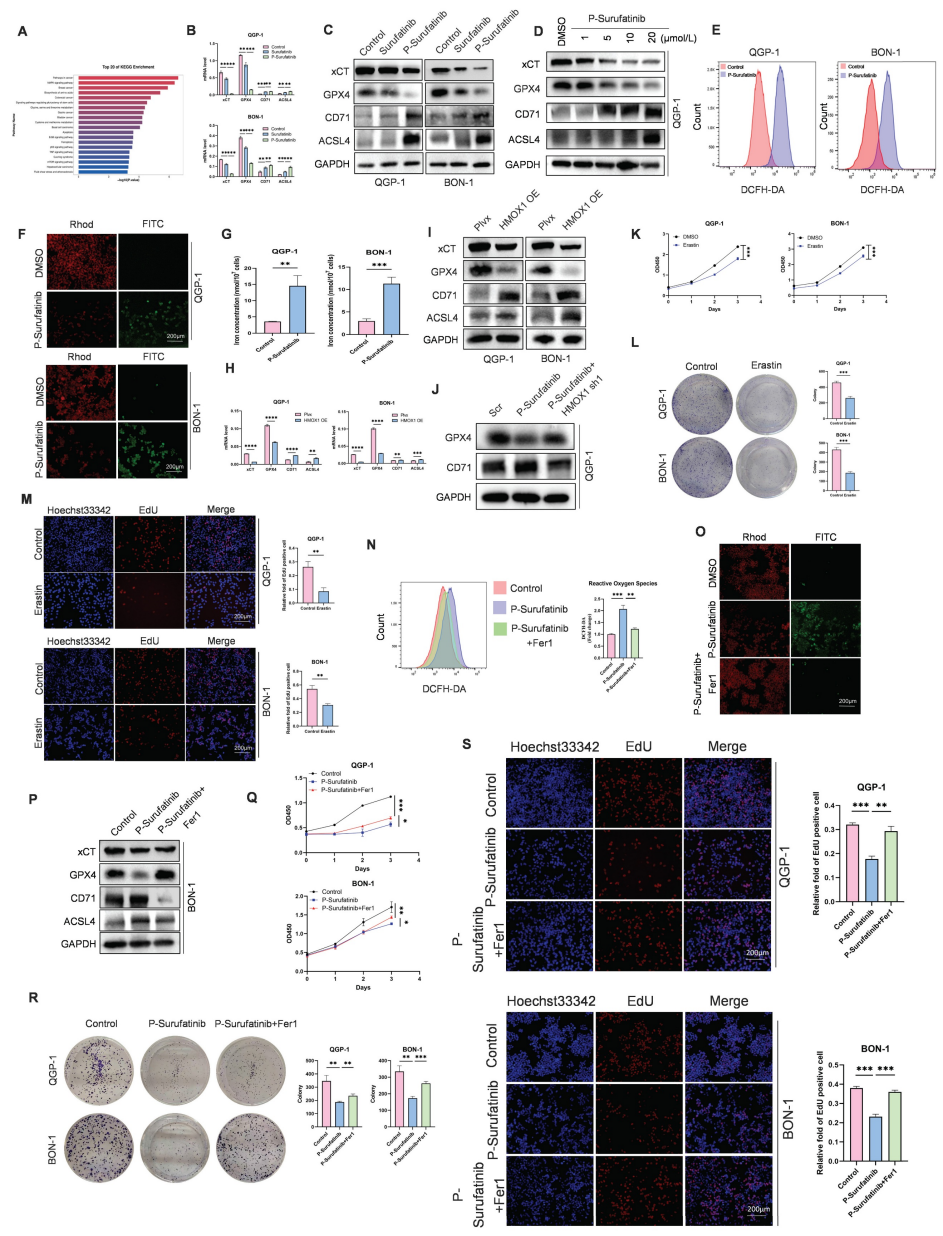
P-Surufatinib suppresses pNENs progression by inducing ferroptosis through upregulation of HMOX1. (A) KEGG enrichment results indicated that P-Surufatinib was involved in many pathways, including ferroptosis. (B, C) qRT-PCR and WB were performed to identify key molecules involved in ferroptosis, including xCT, GPX4, ACSL4, and CD71, in pNENs cells treated with IC_50_ of Surufatinib and P-Surufatinib for 48 h. (D) QGP-1 cells were treated with the indicated concentrations of P-Surufatinib (0μM,1μM,5μM,10μM,20μM) for 48 h. Cells were then harvested for WB of ferroptosis markers. (E) Effect of P-Surufatinib treatment on ROS levels within pNENs cells. (F) Lipid peroxidation assay was performed to explore whether P-Surufatinib induced lipid peroxidation in pNENs cells. Magnification: 200×. (G) Intracellular Fe^2+^ level was determined using kit assays. (H, I) The ferroptosis related biomarkers were evaluated by qRT-PCR and WB in pNENs cells with HMOX1 overexpression. (J) The knockdown of HMOX1 could reverse the activation of ferroptosis mediated by P-Surufatinib. (K-M) CCK-8, colony formation, and EdU incorporation assays were performed to detect whether erastin (10μm for 48h) affects the proliferation of pNENs cells. Magnification: 200×. (N) ROS levels were labeled using DCFH-DA probe and detected by flow cytometry to explore whether ferrostatin-1 (Fer1, 10μm for 48h) could reverse the anticancer function of P-Surufatinib. (O) Lipid peroxidation assay was performed to explore whether Fer1 could reverse the lipid peroxidation induced by P-Surufatinib. Magnification: 200×. (P) Effect of the Fer1 on xCT, GPX4, ACSL4 and CD71 levels of cells treated with P-Surufatinib for 48 h. (Q-S) CCK-8, colony formation, and EdU incorporation assays showed that Fer1 could rescue P-Surufatinib-induced pNENs cell death. Magnification: 200×. *P < 0.01, **P < 0.01, ***P < 0.001, ****P < 0.0001.

According to the transcriptomic outcomes, P-Surufatinib can enhance the cellular death via inducing ferroptosis. Therefore, whether P-Surufatinib could induce ferroptosis in pNENs cells was investigated (Fig. 5A). qRT-PCR and WB were applied to detect the expressional levels of essential molecules during regulating ferroptosis. It was found that xCT and GPX4 expression levels were dramatically decreased while ACSL4 and CD71 expression levels were increased in QGP-1 and BON-1 cells in a dose-dependent pattern in the P-Surufatinib group in comparison with the control group (Fig. 5B-D), which further confirmed that P-Surufatinib could induce ferroptosis.

The ferroptosis-induced cellular death is characterized by the accumulation of ROS and LPO. Therefore, ROS and LPO levels were detected in pNENs cells. ROS level is a key factor for P-Surufatinib-induced cellular death, thus DCFH-DA probe was applied to detect ROS level, and proved that P-Surufatinib could produce a high level of ROS in pNENs cells (Fig. 5E). A LPO experiment revealed that red fluorescent intensity was obviously decreased, and the green fluorescent intensity was increased in QGP-1 and BON-1 cells after P-Surufatinib treatment, which indicated that P-Surufatinib could induce LPO in pNENs cells (Fig. 5F). Iron level measurement also proved that P-Surufatinib could induce cellular iron overload (Fig. 5G). The ferroptosis-related biomarkers such as xCT, GPX4, ACSL4 and CD71 were detected using qRT-PCR and WB, and the results revealed that the overexpressed HMOX1 could obviously decrease the expressional levels of xCT and GPX4, and increase the expressional levels of ACSL4 and CD71 (Fig. 5H, I). Moreover, knock-downing the expression of HMOX1 could reverse the activation of ferroptosis induced by P-Surufatinib (Fig. 5J).

In addition, the pNENs cells were treated with erastin, which could induce ferroptosis to suppress the proliferation of pNENs cells (Fig. 5K-M). BON-1 cells underwent ferrostatin-1 treatment, which could inhibit the ferroptosis. It was found that the tumor-suppressive effect of P-Surufatinib was suppressed to varying extents when the ferroptosis was inhibited (Fig. 5N-P). CCK-8, colony formation, and EdU incorporation assays showed that Fer1 could rescue P-Surufatinib-induced pNENs cell death (Fig. 5Q-S). In general, the above findings suggested that the P-Surufatinib will inevitably lead to ferroptosis, thus further contributing to the suppression of pNENs cell proliferation.

### P-Surufatinib can suppress the WNT/β-catenin signaling pathway via up-regulating the expression of HMOX1 to induce ferroptosis in pNENs cells

**Figure 6:**
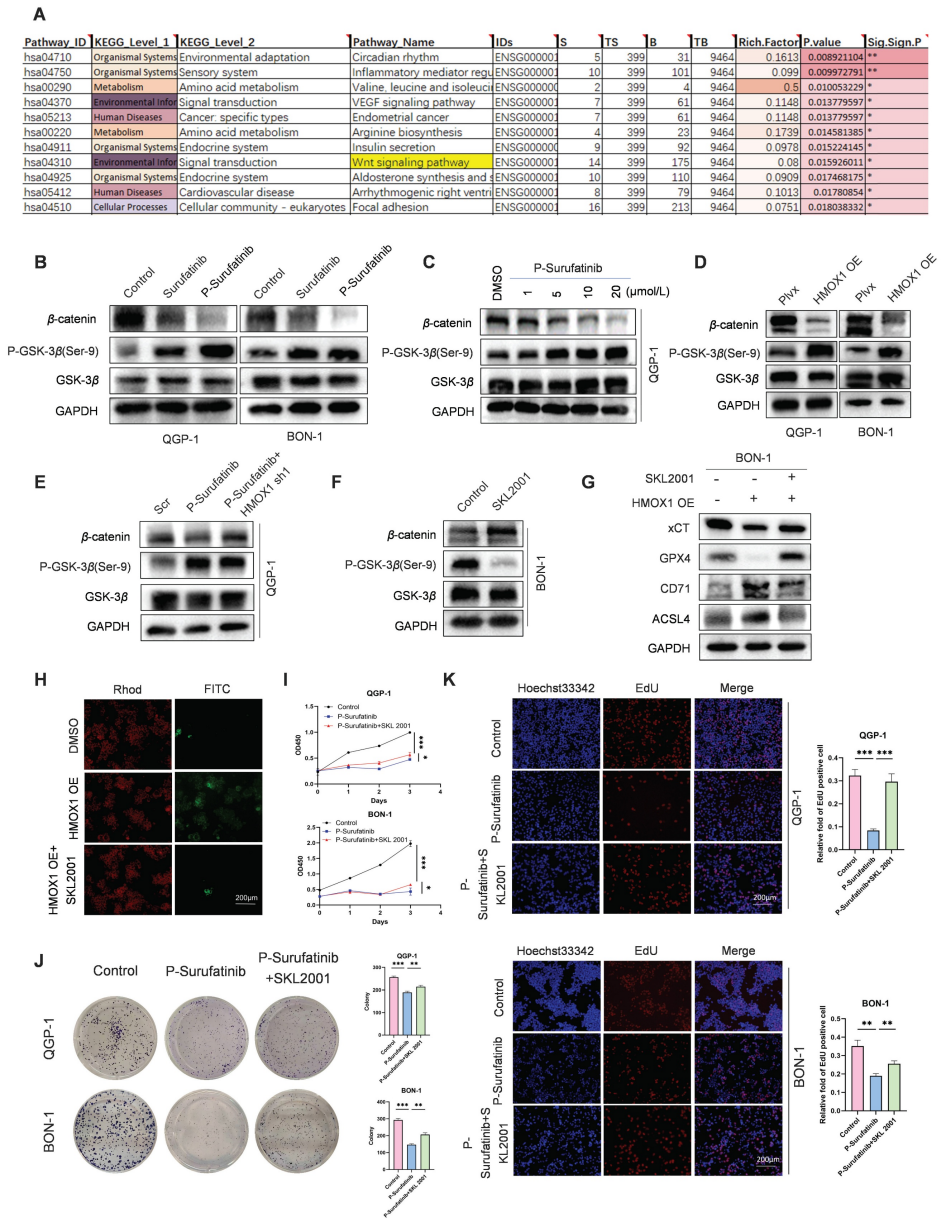
P-Surufatinib suppresses the WNT/β-catenin pathway by upregulating HMOX1 and then induces ferroptosis in pNENs. (A) KEGG enrichment results indicated that P-Surufatinib was involved in many pathways, including the WNT/β-catenin pathway. (B) Key molecules of the WNT/β-catenin pathway, including P-GSK-3β (Ser-9), GSK-3β, and β-catenin, were examined using WB in pNENs cells treated with Surufatinib and P-Surufatinib. (C) After treatment with different concentrations of P-Surufatinib (0μM,1μM,5μM,10μM,20μM), the expression of WNT/β-catenin signaling related biomarkers in QGP-1 cells was tested by WB. (D) The WNT/β-catenin signaling related biomarkers were evaluated by western blot in pNENs cells with HMOX1 overexpression. (E) Key molecules of WNT/β-catenin signaling were examined using WB in QGP-1 cells treated with P-Surufatinib and P-Surufatinib with HMOX1 knockdown. (F) WNT/β-catenin pathway was activated after SKL2001-treated. (G) The ferroptosis related biomarkers were evaluated by WB in overexpression of HMOX1 BON-1 cells treated with SKL2001 (10μM, 48h). (H) SKL2001 suppressed LPO in overexpression of HMOX1 BON-1 cells. Magnification: 200×. (I-K) CCK-8, colony formation, and EdU incorporation assays showed that SKL2001 could reverse P-Surufatinib-induced pNENs cell death. Magnification: 200×. *P < 0.01, **P < 0.01, ***P < 0.001.

KEGG pathway analysis revealed that P-Surufatinib exerts its effects through multiple signaling pathways such as WNT/β-catenin signaling pathway (Fig. 6A). Thereafter, it was proved that P-Surufatinib could inhibit the WNT/β-catenin signaling pathway in a dose-dependent pattern by detecting the biomarkers (P-GSK-3β, GSK-3β and β-catenin) of WNT signaling (Fig. 6B, C). Moreover, it was showed that up-regulated HMOX1 expression could suppress the WNT/β-catenin signaling pathway and knock-downing HMOX1 expression could reverse the inhibitory effect of P-Surufatinib (Fig. 6D, E).

To further verify whether the overexpressed HMOX1 could induce ferroptosis through the WNT/β-catenin signaling pathway, the HMOX1-overexpressed pNENs cells were treated using SKL2001, a novel agonist of WNT/β-catenin signaling. It was revealed that HMOX1-overexpressed pNENs cells exhibited reduced ferroptosis after SKL2001 treatment (Fig. 6F-H). CCK-8, colony formation, and EdU incorporation assays showed that SKL2001 could reverse P-Surufatinib-induced pNENs cell death (Fig. 6I-K). The above findings obviously suggested that HMOX1 could regulate the WNT/β-catenin signaling pathway and further induce ferroptosis in pNENs cells.

### P-Surufatinib can abrogate pNENs cell growth *in vivo* through ferroptosis accumulation

**Figure 7:**
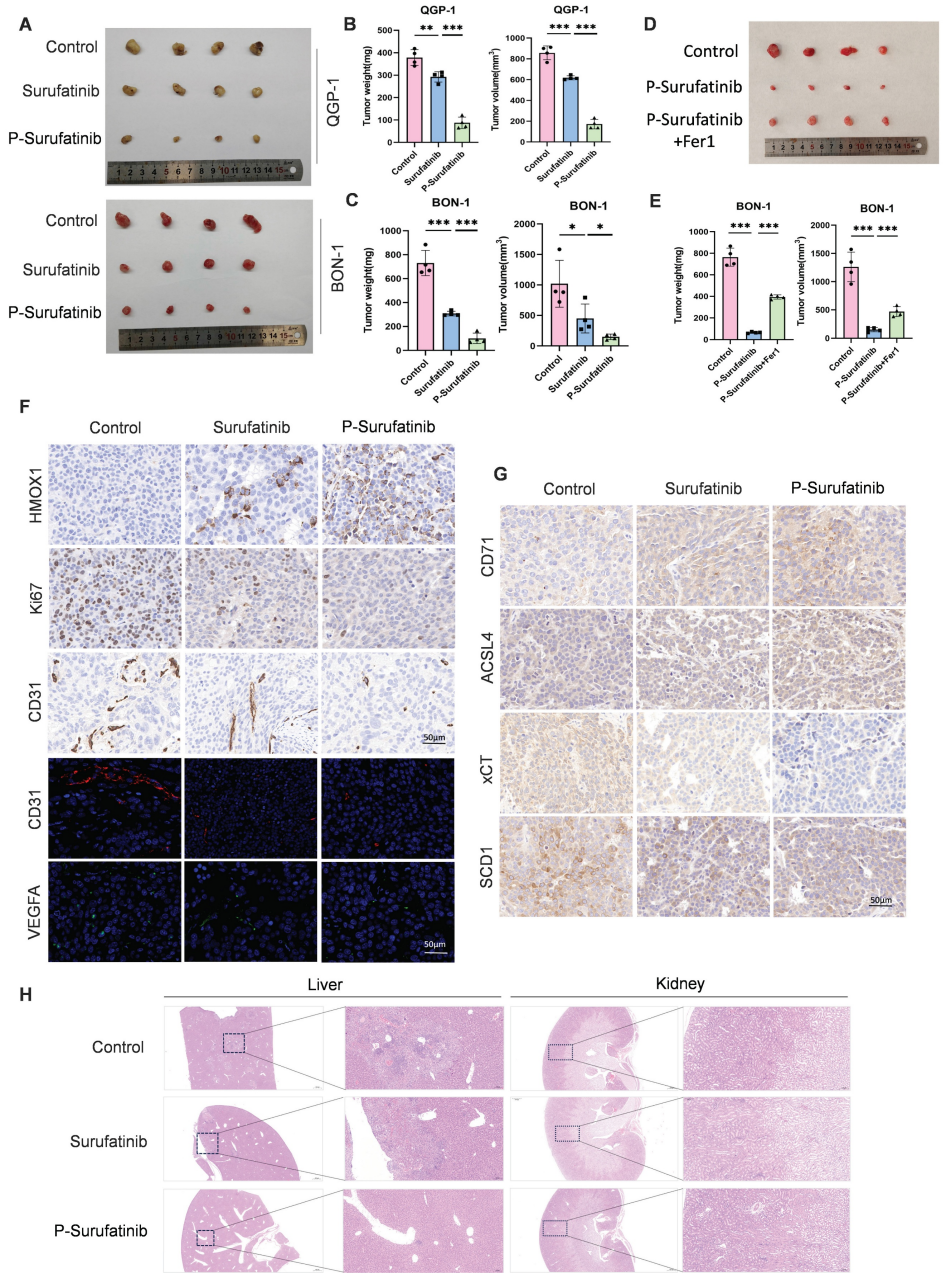
P-Surufatinib abrogates pNENs progression *in vivo* through ferroptosis accumulation. (A) Representative images of xenografted tumors in mice after 14 days following treatment with Surufatinib and P-Surufatinib. (B, C) Tumor weight and volume at the end of treatment. (D) Representative images of xenografted tumors in mice demonstrated the effect of combined treatment with P-Surufatinib and the ferroptosis inhibitor Fer1 on tumor growth. (E) Tumor weight and volume at the end of treatment. (F) The expression of HMOX1, Ki67, CD31, and VEGFA was analyzed by IHC staining. Magnification: 200×. (G) The expression of ferroptosis markers including CD71, ACSL4, xCT, and SCD1 was analyzed by IHC staining. Magnification: 200×. (H) Pathological analysis of liver and kidney tissues of nude mice in Surufatinib and P-Surufatinib group after treatment. *P< 0.01, **P < 0.01, ***P < 0.001.

A nude mouse xenograft model was established to investigate whether P-Surufatinib could suppress the progression of pNENs *in vivo*. It was also found that the tumors in the Surufatinib group had a lower volume and weight compared with the control group, while the tumors in P-Surufatinib group had a lower volume and weight compared with the Surufatinib group (Fig. 7A-C). Furthermore, a nude mouse xenograft model combining treatment with P-Surufatinib and Fer-1 was established. It was found that the tumor-suppressive effect of P-Surufatinib could be suppressed when the ferroptosis was inhibited by Fer-1 *in vivo* (Fig. 7D, E). Immunohistochemistry staining showed that P-Surufatinib up-regulated the expressional level of HMOX1 and down-regulated the expressional levels of Ki-67, CD31 and VEGFA (Fig. 7F). The expression of ferroptosis markers was also analyzed by IHC staining, it was showed that xCT and SCD1 expression levels were dramatically decreased while CD71 and ACSL4 expression levels were increased (Fig. 7G). H&E staining results showed that in P-Surufatinib group, no obvious damage was observed in major organs (Fig. 7H). To sum up, the above findings indicated that P-Surufatinib could reduce the progression and angiogenesis of pNENs* in vivo*.

## Discussion

A lot of studies have indicated that PROTAC can degrade various target proteins such as estrogen receptor (ER)^23^, and androgen receptor (AR) in cancers^24^, with good curative effects on a variety of diseases. The oral PROTACs such as ARV-110 and ARV-471 (ClinicalTrials.gov Identifier: NCT03888612, NCT04072952) were used to degrade AR and ER, respectively, which showed remarkable treatment effects in preclinical studies. Surufatinib has a good anti-tumor effect in the treatment of patients with pNENs, its inevitable adverse reactions include hypertension, albuminuria and liver and kidney injury^7^, ^12^-^14^. In this study, it was firstly tried to enhance the targeting ability of Surufatinib via TK degradation and thus develop a potent TK degrader "P-Surufatinib′′, which had a more superior targeting ability and a higher safety against pNENs. However, the anti-tumor activity of P-Surufatinib and its mechanism of action on pNENs remain unknown. Therefore, this study was conducted and four major findings were concluded. Firstly, it was found that P-Surufatinib can significantly reduce proliferation and angiogenesis of pNENs cells and degrade target proteins. Secondly, P-Surufatinib treatment can obviously reduce the weight and volume of tumors in mice compared with Surufatinib. Thirdly, RNA-seq assays revealed that P-Surufatinib can suppress pNENs progression through the ferroptosis pathway. Fourthly, P-Surufatinib can induce ferroptosis via up-regulating the expression of HMOX1 mediated by WNT/β-catenin signaling pathway. In general, it is firstly reported that P-Surufatinib can decelerate pNENs progression by inducing ferroptosis via up-regulating the expression of HMOX1. GPX4 inhibition-mediated ferroptosis are commonly seen, the mechanistic analysis in this study has proved that the P-Surufatinib can also induce ferroptosis via up-regulating the expression of HMOX1 by suppressing the WNT/β-catenin signaling pathway^25^.

Under the typical condition, HMOX1 has a catalyzing effect on breakdown of heme, resulting in the release of iron, the linear tetrapyrrole biliverdin and carbon monoxides. These byproducts exhibit strong antioxidant properties^26^. Highly-induced HMOX1 can facilitate the breakdown of heme to produce a large amount of free iron, which can further enhance the production of ROS and promote LPO. Therefore, HMOX1 can dually regulate iron and ROS homeostasis, and exert a major effect on ferroptosis^19^, ^27^. Furthermore, HMOX1 has been reported in association with the inactivation of multiple signaling pathways such as AKT/GSK3β and WNT/β-catenin signaling pathways^28^. In this study, P-Surufatinib enhanced a higher expressional level of HMOX1. Then overexpressed HMOX1 could suppress the proliferation of pNENs cells, inhibit the WNT/β-catenin signaling pathway and further induce ferroptosis. Furthermore, knocking down the expressional level of HMOX1 could reverse the effects of P-Surufatinib in suppressing cell proliferation, inhibiting WNT/β-catenin signaling pathway and inducing ferroptotic cellular death in pNENs cells. Overall, HMOX1 can exert a key effect in regulating ferroptosis. Thus, the findings of this study indicate that inducing the overexpression of HMOX1 may be a mechanism for P-Surufatinib to inhibit the proliferation in pNENs cells.

Excessive iron, which plays crucial biological functions, can also have a toxicity due to its ability to produce ROS and trigger cellular death^29^. Recent studies have identified ferroptosis as a regulated form of cellular death associated with tumor development in various carcinomas such as pancreatic, mammary and hepatic carcinomas^30^-^35^. The Fenton reaction can oxidize the labile iron pool (Fe^2+^) into Fe^3+^directly, produce highly reactive hydroxyl radicals, and thus facilitate LPO^22^. The accumulation of LPO ultimately leads to ferroptotic cellular death. Evidence has shown that ROS buildup contributes to ferroptosis in tumors, neurological disorders and injuries^36^-^38^. Therefore, elevated ROS and LPO levels serve as critical markers for ferroptotic cellular death. This study has revealed a significant increase in these markers in P-Surufatinib group.

GPX4 serves as a key ferroptosis regulator, safeguarding cells from this form of cellular death by removing phospholipid peroxides^21^. The study by Zou *et al.* indicated that GPX4 significantly can enhance the sensitivity of clear cell renal cell cancer and ovarian cancer to ferroptosis^39^. Knock-downing the expression of GPX4 can lead to ferroptotic cellular death and increased lipid ROS^40^. Furthermore, the erastin can enhance the ferroptosis, thus is often used in combination with multiple various chemotherapeutic agents such as cisplatin, temozolomide, and adriamycin in the treatments of cancers^41^. This study has investigated whether P-Surufatinib can trigger ferroptosis in pNENs, and the transcriptomic analyses have suggested that P-Surufatinib can mediate the cellular death via ferroptosis pathway. Our findings reveal that P-Surufatinib treatment decreases the mRNA and protein expressional levels of xCT and GPX4 in pNENs cells, and increases the mRNA and protein expressional levels of CD71. In addition, the flow cytometric analysis has indicated that P-Surufatinib can lead to increased ROS level in pNENs cells. The LPO assay has confirmed that P-Surufatinib can induce LPO in pNENs cells. In general, the above findings proved that P-Surufatinib can induce ferroptosis in pNENs *in vitro*. To more accurately assess the extent of ferroptosis in pNENs cells after P-Surufatinib treatment, a reverse experiment was conducted using ferrostatin-1 to inhibit the ferroptosis, which significantly reversed the anti-cancer effect of P-Surufatinib, suggesting that the ability of P-Surufatinib to induce ferroptosis plays a substantial role in its anti-cancer efficacy. Nonetheless, further studies need to be performed in the future to elucidate the precise mechanisms through which P-Surufatinib promotes ferroptosis in pNENs cells.

The correlational analysis have suggested that the WNT/β-catenin signaling pathway can exert a suppressing effect in ferroptosis. β-catenin can move into the nucleus to activate the genes that regulate ferroptosis, including GPX4^42^, COX2^43^, SCD1^44^, thereby hindering the ferroptotic process. The study by Wang *et al.* indicated that the activating WNT/β-catenin signaling pathway can reduce the generation of lipid ROS and further inhibit ferroptosis in gastric cancer cells^45^. Ye Z at al published that MEN1 promotes ferroptosis by inhibiting mTOR-SCD1 axis in pNENs^46^. However, currently there has been no investigation into the correlation between WNT/β-catenin signaling pathway and ferroptosis in pNENs, therefore, this study explored this new approach to the treatment of pNENs. KEGG pathway analysis suggested that P-Surufatinib may exert its effects through various signaling pathways. Our findings revealed a significant correlation between P-Surufatinib and WNT/β-catenin signaling pathway. WB experiment showed that the expressional level of β-catenin was decreased, and the expressional level of phospho-GSK-3β was obviously increased after P-Surufatinib treatment. In addition, up-regulated HMOX1 expression led to suppression of the WNT/β-catenin signaling pathway, while knocking down HMOX1 expression could reverse the suppressive effect of P-Surufatinib. Notably, the SKL2001 treatment could obviously reverse the ferroptosis triggered by overexpressed HMOX1. The above findings suggest that P-Surufatinib can deactivate the WNT/β-catenin signaling pathway through up-regulating the expressional level HMOX1 in pNENs cells, and a potential involvement of ferroptosis requires further exploration.

**Figure 8:**
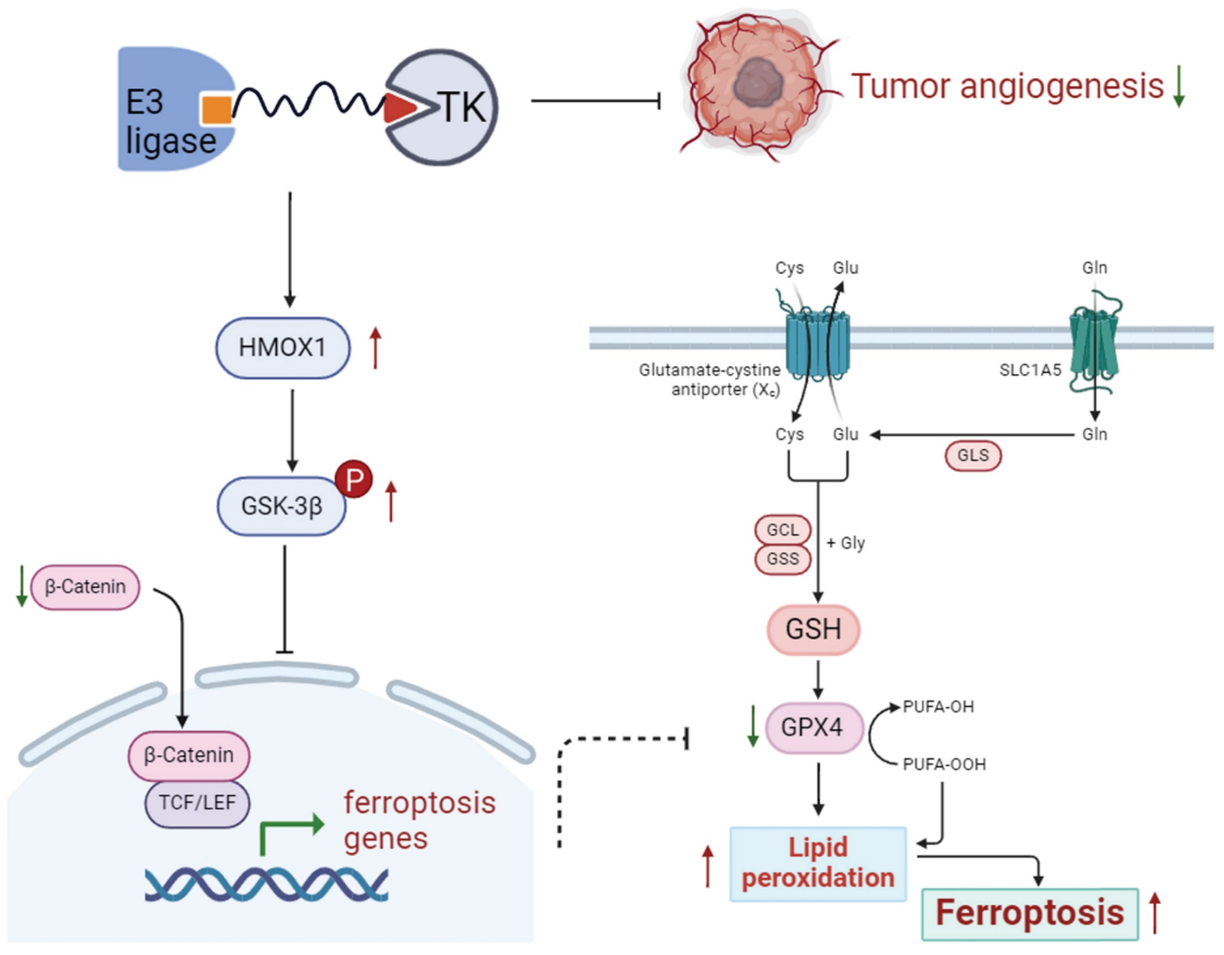
Schematic diagram shows that P-Surufatinib can upregulate a ferroptosis-related gene HMOX1 in pNENs, and the upregulated HMOX1 gene in pNENs can suppress the WNT/β-catenin pathway, which further induces ferroptosis to decrease pNENs cell proliferation and angiogenesis. In addition, we found that knockdown of HMOX1 can reverse the P-Surufatinib-induced suppression of proliferation, as well as the anti-tumorigenic role of HMOX1 in pNENs, which has also been detected *in vitro* and* in vivo*.

In conclusion, as a new cellular death pathway, ferroptosis has become a hot spot in cellular death research, thus having a potential therapeutic value for cancers. The findings of the study confirm that P-Surufatinib can induce pNENs cells to undergo ferroptosis through the activation of HMOX1, which is regulated by the WNT/β-catenin signaling pathway (Fig. 8). This suggests a promising new strategy for combating pNENs and other possible threatening cancers. In the future studies, we aim to further elucidate the molecular mechanism underlying P-Surufatinib-induced ferroptosis.

## Supplementary Material

Supplementary figures and tables.

## Figures and Tables

**Table 1:** The IC_50_ values of Surufatinib and compounds P-Surufatinib 1-3 in pNENs cell lines.

Compound	Constitutional formula	IC_50_(μM)	
		QGP-1	BON-1
Surufatinib	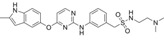	26.83	30.97
P-Surufatinib 1	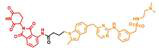	16.36	24.65
P-Surufatinib 2	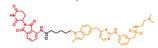	43.81	47.66
P-Surufatinib 3	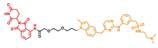	50.74	56.28

**Table 2:** The Dc_50_ values of Surufatinib and P-Surufatinib in QGP-1 cells.

Dc_50_(μM)	Surufatinib	P-Surufatinib
CSF-1R	55.08	4.806
FGFR1	48.43	1.977
VEGFR 1	56.7	8.493
VEGFR 2	57.81	3.975
VEGFR 3	16.29	9.72

## Abbreviations

NENs: Neuroendocrine neoplasms
pNENs: pancreatic neuroendocrine neoplasms
PROTAC: proteolysis-targeting chimera
TK: tyrosine kinase
TKI: tyrosine kinase inhibitor
HMOX1: hemoxygenase 1
VEGFR: vascular endothelial growth factor
FGFR1: fibroblast growth factor 1
CSF1R: colony-stimulating factor 1 receptor
GPX4: glutathione peroxidase 4
CD71: transferrin receptor protein 1
ACSL4: acyl-CoA synthetase long-chain family member 4
IC_50_: Half-maximal inhibitory concentration
CCK-8: Cell counting kit-8
EdU: 5-Ethynyl-2'-deoxyuridine
qRT-PCR: Quantitative real-time polymerase chain reaction
WB: Western blotting
LPO: lipid peroxidation
ROS: reactive oxygen species
Fer-1: ferrostatin-1
KEGG: Kyoto Encyclopedia of Genes and Genomes
GO: Gene ontology
IHC: Immunohistochemistry
GAPDH: Glyceraldehyde-3-phosphate dehydrogenase
