# Supplementary material for: PROTAC-Surufatinib Suppresses Pancreatic Neuroendocrine Neoplasms Progression by Inducing Ferroptosis through Inhibiting WNT/β-catenin Pathway Mediated by HMOX1
Source: Int J Biol Sci. 2025 Mar 19;21(6):2476–92. doi: 10.7150/ijbs.106357 (PMC12035883; doi:10.7150/ijbs.106357)

Table S1 Short hairpin targets

Gene names	Target sequence(5'-3')
HMOX1 sh1	ACAGTTGCTGTAGGGCTTTAT
HMOX1 sh2	GCTGAGTTCATGAGGAACTTT

Table S2 Primers of genes

Gene names	Sequence(5'-3')
HMOX1 forward	AAGACTGCGTTCCTGCTCAAC
HMOX1 reverse	AAAGCCCTACAGCAACTGTCG
xCT forward	TCTCCAAAGGAGGTTACCTGC
xCT reverse	AGACTCCCCTCAGTAAAGTGAC
CD71 forward	ACCATTGTCATATACCCGGTTCA
CD71 reverse	CAATAGCCCAAGTAGCCAATCAT
GPX4 forward	GAGGCAAGACCGAAGTAAACTAC
GPX4 reverse	CCGAACTGGTTACACGGGAA
ACSL4 forward	CATCCCTGGAGCAGATACTCT
ACSL4 reverse	TCACTTAGGATTTCCCTGGTCC
ACTB forward	CATGTACGTTGCTATCCAGGC
ACTB reverse	CTCCTTAATGTCACGCACGAT
GAPDH forward	GGAGCGAGATCCCTCCAAAAT
GAPDH reverse	GGCTGTTGTCATACTTCTCATGG

Table S3 Antibody information

Antibody	Company	Catalogue
GAPDH	Proteintech	60004-1-Ig
β-Actin	Proteintech	60008-1-Ig
CSF1R	Bioss	bsm-51303M
FGFR1	Proteintech	60325-1-Ig
VEGFR1	Origene	TA384238
VEGFR2	Abcam	ab134191
VEGFR3	Proteintech	20712-1-AP
HMOX1	Abcam	ab68477
xCT	Abmart	T57046
GPX4	Abmart	T56959
CD71	Proteintech	10084-2-AP
ACSL4	Proteintech	22401-1-AP
β-catenin	Proteintech	51067-2-AP
P-GSK-3β(Ser-9)	CST	9323
GSK-3β	Proteintech	22104-1-AP
Goat Anti-Mouse IgG	CWBIO	CW0102S
Goat Anti-Rabbit IgG	CWBIO	CW0103S

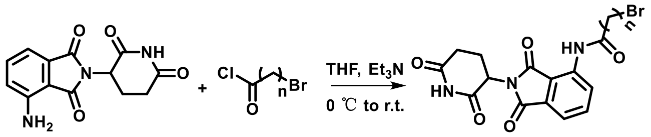

2a-c
n= 1, 3, 5
3a-c

General procedure for the synthesis of pomadomide derivatives 3a-3c.

Pomadomide (3.0 g, 11 mmol) was firstly dissolved in 30 mL anhydrous THF under nitrogen atmosphere. Then, Bromoalkanoyl chloride **2a-2c** (14 mmol) was added into the reaction mixture under nitrogen atmosphere within 1 h at 0 °C. The mixture was further stirred at room temperature for 2 h. Then another 200 mL dichloromethane was added into the mixture, washed three times with deionized water, dried with anhydrous sodium sulfate and evaporated. After that, the crude product was purified by column chromatography (*v*: *v*= Methanol: CH_2Cl_2) to give **3a-3c** as yellow solid.

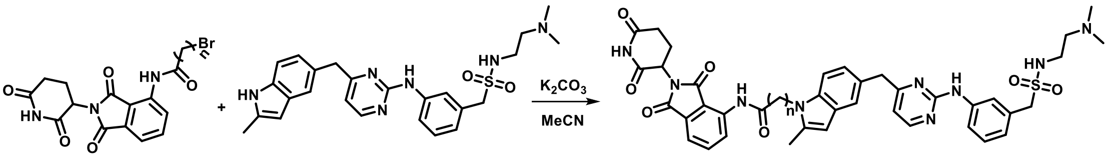

3a-c
4a-c

General procedure for the synthesis of pomadomide derivatives 4a-4c.

Compound **3a-3d** (1 mmol), Sofantinib (0.48 g, 1 mmol) and K_2CO_3 (0.28 g, 2 mmol) was dissolved in 20 mL anhydrous MeCN. The resulting mixture was stirred at 60 °C for another 6 h. The resulting mixture was filtered, and the solvent was removed by evaporation. The crude product was purified by column chromatography (*v*: *v*= Methanol: CH_2Cl_2) to give **4a-4c** as yellow solid.

4-(5-((2-((3-((N-(2-(dimethylamino)ethyl)sulfamoyl)methyl)phenyl)amino)pyrimidin-4-yl)methyl)-2-methyl-1H-indol-1-yl)-N-(2-(2,6-dioxopiperidin-3-yl)-1,3-dioxoisoindolin-4-yl)acetamide (4a)

Yellow solid (42 % yield): ^{1}H NMR (300 MHz, DMSO-*d6*) δ 11.09 (d, *J* = 14.3 Hz, 2H), 9.61 (d, *J* = 9.4 Hz, 1H), 8.31 (dd, *J* = 5.6, 2.1 Hz, 1H), 7.56 (q, *J* = 7.2 Hz, 2H), 7.50 - 7.45 (m, 1H), 7.33 (d, *J* = 8.6 Hz, 1H), 7.21 (d, *J* = 2.3 Hz, 1H), 7.02 (dd, *J* = 7.6, 5.4 Hz, 3H), 6.89 - 6.83 (m, 2H), 6.54 (s, 1H), 6.30 (d, *J* = 5.6 Hz, 1H), 6.15 (s, 1H),

5.06 (dd, J = 12.7, 5.4 Hz, 1H), 4.06 (s, 2H), 3.53 (t, J = 6.7 Hz, 4H), 3.29 (d, J = 5.2 Hz, 2H), 3.05 - 2.87 (m, 4H), 2.64 - 2.46 (m, 5H), 2.29 (t, J = 6.8 Hz, 2H), 2.07 - 1.95 (m, 1H), 1.87 - 1.72 (m, 4H), 1.47 - 1.33 (m, 4H).

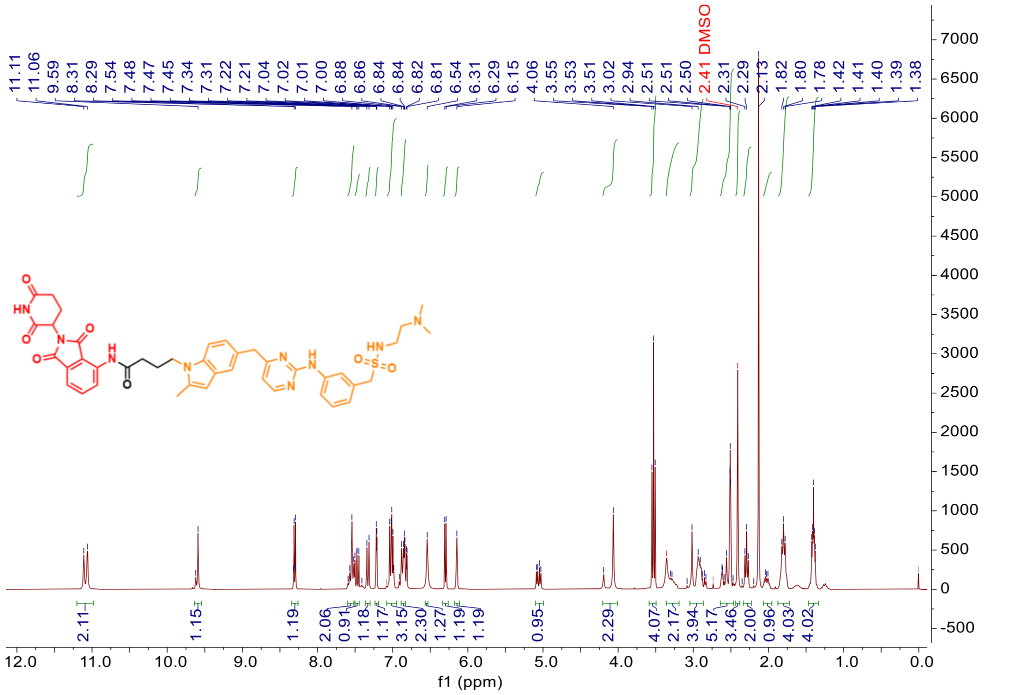

Yellow solid: ^{13}C NMR (101 MHz, DMSO-*d6*) δ 173.3, 171.0, 170.6, 169.1, 167.8, 160.3, 160.0, 147.2, 145.8, 140.8, 137.8, 135.9, 134.3, 132.5, 130.8, 129.6, 128.5, 124.2, 122.2, 121.4, 119.0, 114.6, 111.6, 109.0, 99.9, 98.5, 59.4, 58.0, 49.0, 45.6, 41.2, 31.5, 22.6, 13.9.

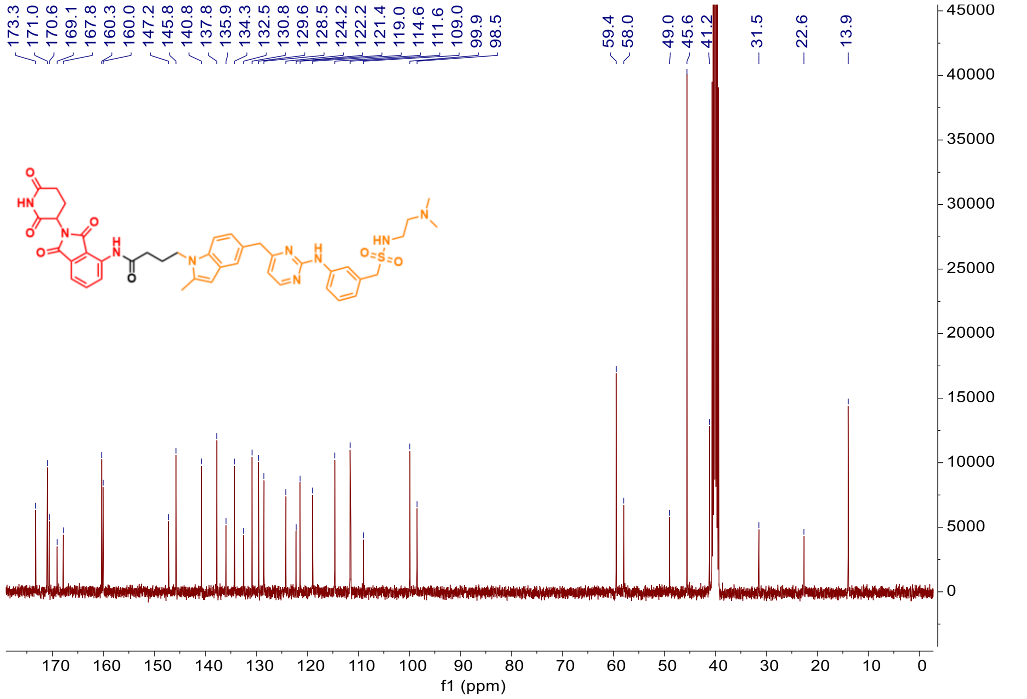

6-(5-((2-((3-((N-(2-(dimethylamino)ethyl)sulfamoyl)methyl)phenyl)amino)pyrimidin-4-yl)methyl)-2-methyl-1H-indol-1-yl)-N-(2-(2,6-dioxopiperidin-3-yl)-1,3-dioxoisoindolin-4-yl)butanamide (4b)

Yellow solid (36 % yield); ^{1}H NMR (300 MHz, DMSO-d6) δ 11.09 (d, J = 15.3 Hz, 2H), 9.59 (s, 1H), 8.30 (d, J = 5.6 Hz, 1H), 7.53 (d, J = 7.8 Hz, 2H), 7.48 (dd, J = 8.4, 7.1 Hz, 1H), 7.33 (d, J = 8.6 Hz, 1H), 7.21 (d, J = 2.2 Hz, 1H), 7.02 (dd, J = 7.6, 5.0 Hz, 3H), 6.90 - 6.77 (m, 3H), 6.54 (s, 2H), 6.30 (d, J = 5.6 Hz, 1H), 6.15 (s, 1H), 5.06 (dd, J = 12.7, 5.4 Hz, 1H), 4.06 (t, J = 7.1 Hz, 3H), 3.55 (t, J = 6.6 Hz, 1H), 3.05 - 2.82 (m, 3H), 2.67 - 2.51 (m, 2H), 2.49 - 2.33 (m, 5H), 2.27 (t, J = 6.8 Hz, 2H), 2.05 (p, J = 6.9 Hz, 3H), 1.19 (t, J = 7.1 Hz, 2H).

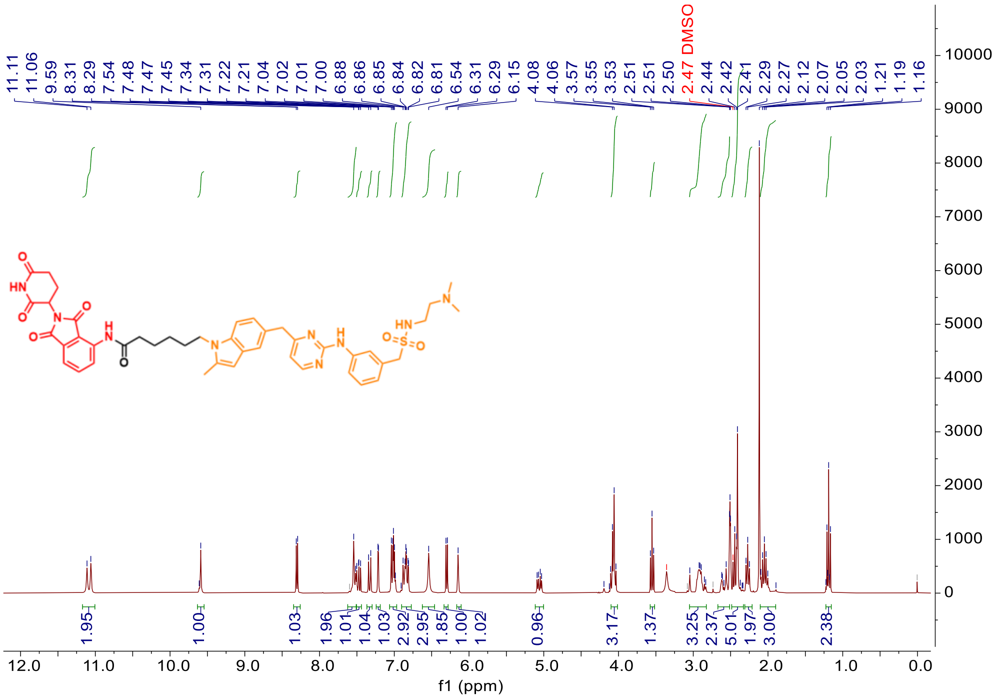

Yellow solid: ^{13}C NMR (101 MHz, DMSO-*d6*) δ 174.9, 173.3, 171.0, 170.6, 169.1, 167.9, 160.3, 160.0, 147.2, 145.8, 140.9, 140.8, 137.8, 135.9, 134.3, 132.5, 130.8, 130.4, 129.6, 128.5, 124.2, 122.2, 121.4, 119.0, 114.6, 111.6, 109.0, 99.9, 98.5, 59.2, 57.9, 51.0, 49.0, 45.4, 41.0, 36.9, 35.5, 35.4, 34.0, 33.8, 32.4, 31.5, 27.6, 25.6, 24.4, 24.1, 22.6, 21.9, 13.9.

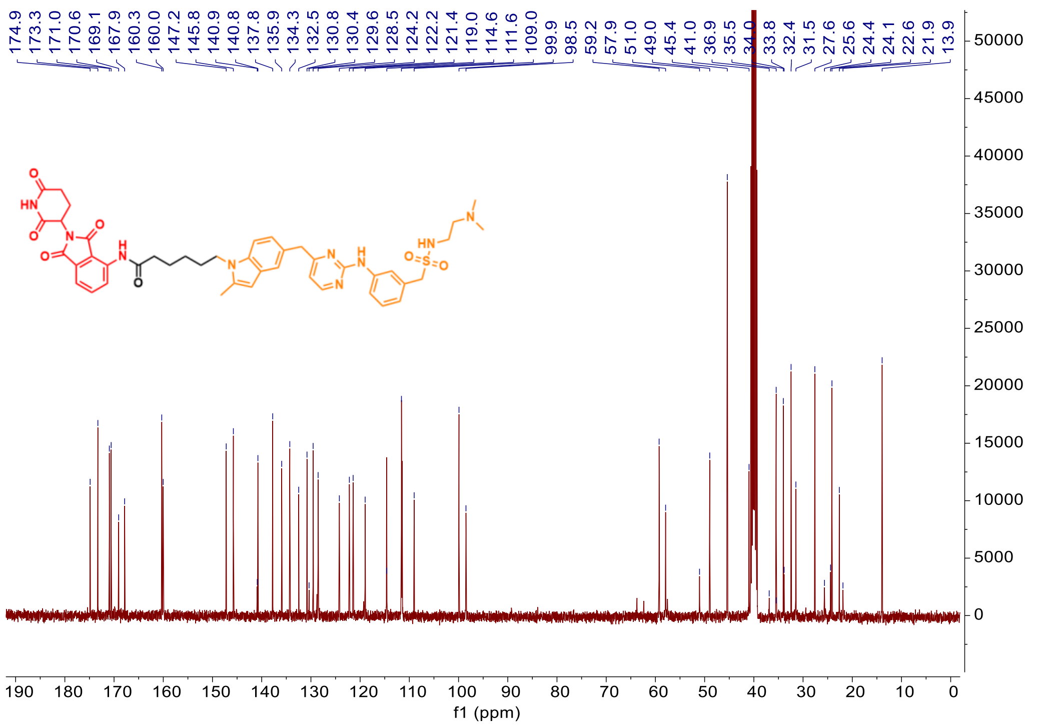

2-(2-(2-(3-(5-((2-((3-((N-(2-(dimethylamino)ethyl)sulfamoyl)methyl)phenyl)amino)pyrimidin-4-yl)methyl)-2-methyl-1H-indol-1-yl)-N-(2-(2,6-dioxopiperidin-3-yl)-1,3-dioxoisoindolin-4-yl)hexanamide (4c)

Yellow solid (40 % yield); ^{1}H NMR (300 MHz, DMSO-*d6*) δ 11.09 (d, *J* = 13.8 Hz, 2H), 9.60 (d, *J* = 8.0 Hz, 1H), 8.31 (dd, *J* = 5.6, 2.1 Hz, 1H), 7.60 - 7.45 (m, 3H), 7.33 (d, *J* = 8.6 Hz, 1H), 7.21 (d, *J* = 2.3 Hz, 1H), 7.02 (dd, *J* = 7.6, 5.1 Hz, 3H), 6.87 (d, *J* = 7.5 Hz, 1H), 6.83 (dd, *J* = 8.6, 2.3 Hz, 1H), 6.54 (s, 2H), 6.30 (d, *J* = 5.6 Hz, 1H), 6.15 (s, 1H), 5.06 (dd, *J* = 12.7, 5.4 Hz, 1H), 4.06 (s, 2H), 3.60 - 3.55 (m, 4H), 3.05 - 2.81 (m, 4H), 2.64 - 2.52 (m, 2H), 2.41 (s, 3H), 2.30 (t, *J* = 6.8 Hz, 2H), 2.14 (s, 3H), 2.03 (dd, *J* = 10.4, 5.2 Hz, 1H), 1.92 (h, *J* = 3.2 Hz, 4H).

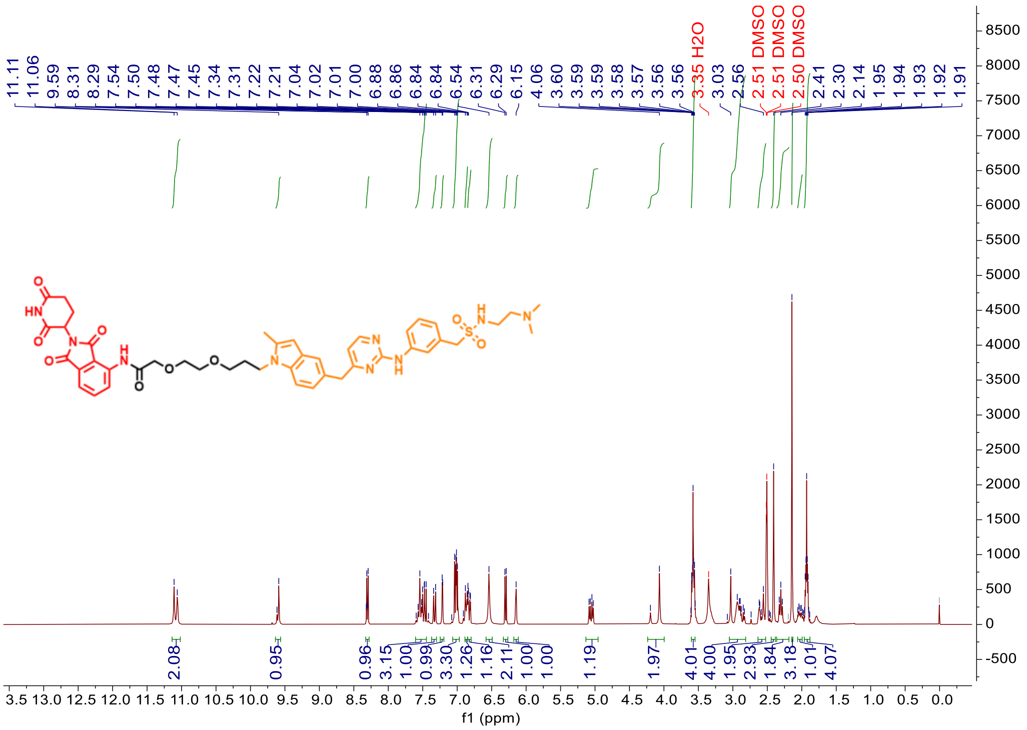

Yellow solid: ^{13}C NMR (101 MHz, DMSO-*d6*) δ 173.3, 171.0, 170.6, 169.1, 160.3, 160.0, 147.2, 145.8, 140.8, 137.8, 135.9, 134.3, 132.5, 130.8, 129.6, 128.5, 124.2, 122.2, 121.4, 119.0, 114.6, 111.6, 111.5, 109.0, 99.9, 98.5, 59.4, 58.0, 49.0, 45.5, 41.1, 31.5, 22.6, 13.9.

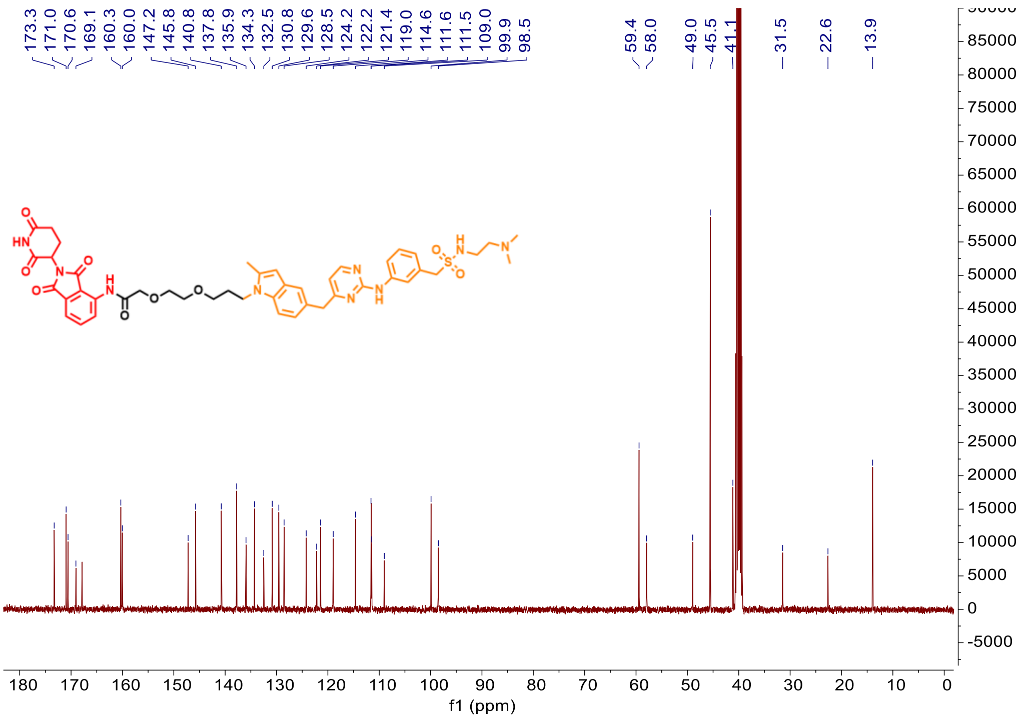

Supplement: Supplementary file 1 — Supplementary figures and tables. [file ijbsv21p2476s1.pdf]
